# Antibacterial activity and effects of *Colla corii asini* on *Salmonella typhimurium* invasion in vitro and in vivo

**DOI:** 10.1186/s12906-017-2020-9

**Published:** 2017-12-04

**Authors:** Kwang-il Park, Mi-ra Lee, Tae-woo Oh, Kwang-Youn Kim, Jin-yeul Ma

**Affiliations:** Korean Medicine (KM)-Application Center, Korea Institute of Oriental Medicine (KIOM), 70 Cheomdan-ro, Dong-gu, Daegu, 41062 Republic of Korea

**Keywords:** *Colla corii asini*, *Salmonella* typhimurium, Antibacterial activity, Cell invasion, Salmonella invasion protein

## Abstract

**Background:**

*Salmonella enterica serovar* Typhimurium is a foodborne pathogen that triggers inflammatory responses in the intestines of humans and livestock. *Colla corii asini* is a traditional medicine used to treat gynecologic and chronic diseases in Korea and China. However, the antibacterial activity of *Colla corii asini* has been unknown. In this study, we investigated the antibacterial activity and effects of *Colla corii asini* extract on *Salmonella typhimurium* invasion.

**Methods:**

To tested for antibacterial effects of *Colla corii asini* extracts, we confirmed the agar diffusion using Luria solid broth medium. Also, we determined the MIC (minimum inhibitory concentration) and the MBC (minimum bactericidal concentration) value of the *Colla corii asini* ethanol extract (CEE) by using two-fold serial dilution methods. We evaluated the expression of salmonella invasion proteins including SipA, SipB and SipC by using Western blot and qPCR at the concentration of CEE without inhibition of bacterial growth. In vitro and *vivo*, we determined the inhibitory effect of invasion of *S.* typhimurium on CEE by using gentamicin assay and *S.* typhimurium*-*infected mice*.*

**Results:**

CEE significantly inhibited the growth of *Salmonella* typhimurium in an agar diffuse assay and had an MIC of 0.78 mg/ml and an MBC of 1.56 mg/ml. Additionally, CEE reduced *Salmonella* typhimurium cell invasion via the inhibition of *Salmonella* typhimurium invasion proteins, such as SipA, SipB and SipC. Furthermore, CEE significantly suppressed invasion in the small intestines (ilea) of mice injected with *Salmonella* typhimurium.

**Conclusion:**

These findings show that *Colla corii asini* exerts antibacterial activity and suppresses *Salmonella* typhimurium invasion in vitro and in vivo. Together, these findings demonstrate that *Colla corii asini* is a potentially useful therapeutic herbal medicine for treating salmonella-mediated diseases.

## Background

*Colla corii asini* (donkey-hide gelatin, E-Jiao) is a well-known traditional Chinese medicine [1] and has been used for more than 2000 years in Asia for health-care food [2]. Previous reports have shown that the main components of *Colla corii asini* comprise amino acids, microelements and small molecular weight hydrolysates of collagen proteins [3]. Some pharmacological properties of *Colla corii asini* include sedation, anticoagulation, vasodilatation, hematopoiesis, as well as the improvement of cellular immunity and radio-protection [3, 4]. It has been widely used for the treatment of gynecologic diseases (e.g., dysmenorrhea, menoxenia, metrorrhagia, abortion) [5] and some chronic diseases (e.g., anxiety, insomnia, apostaxis, hemoptysis, hematuria, hemafecia) [6]. Also, *Colla corii asini* has the anti-aging effect through enhancing antioxidant activity, scavenging free radicals, and modulating aging-related gene expression in aging mice model [7]. Furthermore, *Colla corii asini* promotes anemia and optimizes hemoglobin components in pregnant women with thalassemia [8]. Despite the increasing number of studies on the pharmacological effects of *Colla corii asini*, the antibacterial effect of *Colla corii asini* has not yet been investigated.

The gram-negative bacterium *Salmonella enterica serovar* Typhimurium (*S.* typhimurium) is the main cause of gastroenteritis in humans and animals around the world, and it occurs via bacterial gastrointestinal infections [9]. These bacterial infections are caused by oral intake of contaminated food or water or by fecal contamination [10, 11]. *Salmonella* epidemics associated with contaminated food have been shown to result from insufficient hygiene conditions. *S.* typhimurium is classified as a non-typhoidal serovar, which can infect a broad range of animals and humans and can cause acute intestinal inflammation and diarrhea [12]. The systemic spread of *S.* typhimurium may cause severe disease in people with underlying infections or immunodeficiency disorders and can trigger an enormous economic loss in animal husbandry. *S*. typhimurium relies on two distinct groups of genes, *Salmonella* pathogenicity islands (SPI)-1 and SPI-2, which play pivotal roles in the colonization of the host [13]. SPI-1, which encodes a type III secretion system (T3SS), secretes effectors such as SipA, SipB and SipC, which promote host invasion in the small intestinal tract via the formation of actin filaments at the bacterial entry site [14]. In contrast, *Salmonella* pathogenicity islands 2 (SPI-2) are required for proliferation inside the host cells [15].

In this study, we investigated the antibacterial effects of *Colla corii asini* by using *S.* typhimurium in vitro and in vivo*.* Furthermore, our findings suggest clues that may aid in understanding the antibacterial action of *Colla corii asini* and determining whether *Colla corii asini* may be a good candidate for treating bacterial diseases.

## Methods

### Source of bacterial strain and culture condition

The gram-negative bacterium S. typhimurium KCTC 1926 was obtained from the Korean Research Institute of Bioscience & Biotechnology (KRIBB). For the experiment, bacteria were cultured at 37 °C with shaking in a 5 ml Luria-Bertani (LB) broth medium, until 0.5–0.6 OD value at 600 nm, pelleted at 5000 x g for 10 min, washed in PBS, and resuspended in 1 ml Dulbecco Modified Eagle Medium (DMEM) completed media for cell infection (1 × 10^8^/ml).

### Cell culture

Human colorectal cancer cells, caco-2 cells were maintained in DMEM with 1% antibiotics (penicillin-streptomycin), and 10% fetal bovine serum (FBS) at 37 °C in 10% CO_2_. Cells were seeded at 1 × 10^6^ cells per well in 6-well tissue culture plates containing or not coverslips and maintained as differentiated monolayers for 21–28 days, changing the media every 2–3 days [16].

### Preparation of *Colla corii asini* extract

*Colla corii asini* were purchased from the Korea Medicinal Herbs Association (Yeongcheon, Korea). The identification of *Colla corii asini* was confirmed by Professor KiHwan Bae of the College of Pharmacy, Chungnam National University (Daejeon, Korea), and all voucher specimens were deposited in the herbal bank in the Korea Institute of Oriental Medicine (KIOM, Korea).

To prepare the Colla corii asini ethanol extract (CEE), Colla corii asini (50 g) was grounded to powder, then suspended in 70% ethanol (300 ml) on shaking incubator (100 rpm) for 24 h at 40 °C. The solution was filtered through a nylon net filter (60 μm; Millipore Co., Denver, MA, USA), and then deposited overnight. The supernatant was lyophilized, and then the dried pellet (the yield, 1.66%) was stored at −20 °C until use. Also, to prepared the Colla corii asini water extract (CWE), Colla corii asini (50 g) were placed in 1000 mL distilled water and then extracted during 2 h of heating at 115 °C (Gyeongseo Extractor Cosmos-600, Inchon, Korea), and the solution was filtered using standard testing sieves (140 μm) (Retsch, Haan, Germany). The supernatant was lyophilized, and then the dried pellet (the yield, 38.70%) was stored at −20 °C until use.

### Amino acid content in *Colla corii asini*

The amino acid composition of Colla corii asini were assayed with an L-8900 amino acid analyzer (Hitachi, Japan) after hydrolysis of 20 mg sample with 0.02 N HCl at 110 °C for 24 h. The Aspartic acid (Asp), Threonine (Thr), Serine (Ser), Glutamic acid (Glu), Glycine (Gly), Alanine (Ala), Cysteine (Cys), Valine (Val), Methionine (Met), Isoleucine (Ile), Leucine (Leu), Tyrosine (Tyr), Phenylalanine (Phe), Lysine (Lys), Histidine (His), Arginine (Arg), Proline (Pro), Valine (Val), β-alanine and ethanol amine content were quantified by standard peak values.

### Agar well diffusion assay

The extracts were tested for antibacterial effects by using agar diffusion on Luria agar [17]. The solid agar was punched with 7 mm diameter holes using the steriled tips. The agar plates included 1% S. typhimurium and were then treated with 100 μl of Colla corii asini extract. The concentrations of the extracts (EtOH and water) applied were 0.25–2 mg/ml, and ampicillin (5–40 μg) was used as a positive control. The solid agar was then incubated at 37 °C for 24 h. After incubation, the clear zone associated with each concentration was measured.

### Determination of the minimum inhibitory concentration and minimum bactericidal concentration

To determine the MIC (minimum inhibitory concentration) of the CEE, we detected the lowest concentration that did not demonstrate visible bacterial growth after 24 h of incubation at 37 °C. The MIC was determined by using two-fold serial dilutions of the extracts [18]. The MBC (minimum bactericidal concentration) value was determined by sub-culturing the test dilutions on a solid medium and incubating the cultures at 37 °C for 24 h. The MBC value indicated the lowest dilution concentration at which there was no bacterial growth on the solid medium.

### Analysis of protein secretion

*S.* typhimurium (1 × 10^8^ cells) was incubated in 6-well plates. CEE was treated to S. typhimurium for 1 h, and then collected the DMEM media, and centrifuged, and the supernatants (2 ml) were collected. The supernatants were filtered (0.45-μm pore size), and the proteins were precipitated with 100% trichloroacetic acid (TCA) by using high-speed centrifugation (14,000×g for 30 min). The pellet was washed in cold acetone and resuspended in PBS. The solutions contained proteins and were mixed with Sodium Dodecyl Sulphate (SDS) sample buffer and boiled for 5 min. Each sample was separated by SDS-10% polyacrylamide gel electrophoresis (SDS-PAGE). The expression of SipC protein were analyzed by Western blot. Bacteria were lysed using ice-cold lysis buffer, and the protein concentration were determined by bicinchoninic acid (BCA) assay. Equal amounts of protein (30 μg/mL) were electrophoresed on 4–10% SDS-acrylamide gels and transferred to nitrocellulose membranes using an electric transfer system. Blots were incubated for overnight at 4 °C with primary antibody against anti-SipC (1:100; ABIN335178). After three times washes with TBS-T, the membranes were incubated with HRP conjugated goat anti-mouse IgG (1:2000) for 1 h at room temperature. The signals were detected with a chemiluminescence reagent (Millipore Corporation, Billerica, USA), and analyzed with the ChemiDoc Touch Imaging System (Bio-Rad, Hercules). Protein bands were quantified by densitometry using Image J

### Quantitative real-time PCR

*S.* typhimurium (1 × 10^8^ cells) was incubated in 6-well plates. CEE was treated to S. typhimurium for 1 h and mRNA was purified using TRIzol reagent and chloroform. Reverse transcription was conducted in 20 μl reactions with 1 μg of total RNA transformed into cDNA using AccuPower Cycle Script RT premix (Bioneer). The measurements of SipA, SipB and SipC mRNA and 16 s rRNA were conducted under the following conditions: 45 cycles of 95 °C for 10 s, 60 °C for 20 s, and 72 °C for 30 s using a LightCycler 480 II (Roche, Rotkreuz, SWI). The mRNA level in each sample was quantified on the basis of the threshold cycle (Ct). The target genes were normalized relative to the reference gene 16S rRNA [19].

### Cell viability

Cell viability was determined by using a Cell Counting Kit-8 (CCK-8) according to the manufacturer’s instructions by utilizing Dojindo highly water-soluble tetrazolium salt (Dojindo Molecular Technologies, Inc., Rockville, MD, USA). [2-(2-methoxy-4-nitrophenyl)-3-(4-nitrophenyl)-5-(2,4-disulfophenyl)-2H–tetrazolium, monosodium salt] (WST-8) was reduced by dehydrogenases in cells, to forming a yellow product (formazan) generated by the activity of dehydrogenases in cells in a manner directly proportional to the number of live cells. Briefly, Caco-2 cells (1 × 10^3^ cells) were plated on a 96-filter plate. After CEE treatments (1.56–0.04875 mg/ml) for 24 h, 10 μl of the CCK-8 solution was added to each well of the plate and incubated for 1 h at 37 °C with 5% CO2. Absorbance was measured at 450 nm using a precision microplate reader (Molecular Devices, Sunnyvale, CA, USA).

### Selectivity index (SI)

SI was calculated dividing the IC_50_ value by the MIC value for CEE [20, 21].

### Gentamicin assay

*S.* typhimurium was used to infect Caco-2 cells (1 × 10^6^ cell/well) in in 6 well plate. CEE was used to treat Caco-2 cells with/without bacterial infection (50 multiplicity of infection (MOI)) in antibiotic-free medium, non-adherent bacteria were washed three times by PBS. Gentamicin (100 μg/ml) was then added for 1 h to kill any remaining bacteria. The Caco-2 cells were washed three times with PBS and lysed with 1% Triton-X-100. Bacteria in the lysis solutions were plated on solid agar, and colony numbers were counted after O/N incubation at 37 °C [22].

### Infection of *Salmonella enterica* serovar Typhimurium in mice

The infection of S. typhimurium was initiated in 6 to 7-week-old male ICR mice (Samtako Inc., Gyunggi-do, Korea). All mice were divided into 4 groups, each consisting of 5 mice. Mice were administered Colla corii asini extract (100 or 200 mg/kg) via oral gavage daily. Mice were administered either 100 μl of S. typhimurium (5 × 10^7^ cells) or a vehicle control (PBS) by gavage. Forty-eight hours post-infection, mice were euthanized, and tissues (small intestine) were obtained from each treatment and the control group. Intestine samples were stored using the “Swiss roll” technique for immunohistochemistry (IHC) analysis [23]. All procedures were approved by the Korea Institute of Oriental Medicine Institutional Animal Care and Use Committee (KIOM-IACUC) and were conducted in accordance with the US guidelines (NIH publication #83–23, revised in 1985).

### Histological analysis

After tissue fixation, Swiss rolled intestinal tissues were embedded in paraffin and were sectioned into 5 μm sections using a microtome. The serial sections were subjected to immuno-fluorescence staining using fluorescence-tagged antibodies for measuring the number of *S. typhimurium* in the small intestine (ileum) using a confocal microscope.

### Statistical analysis

For analysis of the statistical significance of differences between two groups we generally performed two-tailed Student’s t-tests. For analysis of the statistical significance of differences among more than two groups, we performed one-way ANOVAs with Dunnett’s multiple comparisons tests. All statistical analysis was performed using GraphPad Prism version 5.03 for Windows (GraphPad Software Inc., San Diego, CA, USA).

## Results

### Content of amino acids in *Colla corii asini*

Table 1 shows that CEE contained the major amino acids. Glycine (8.639 mg/g) has been the highest amino acid content in CEE. The total free amino acids content was 21.662 mg/g.

**Table 1:** Contents of Amino Acids in *Colla corii asini*

	EtOH extract	Water extract
Amino acid	mg/g	
Glycine (Gly)	8.639	0.208
Alanine (Ala)	2.527	0.062
Phenylalanine (Phe)	0.586	0.005
Leucine (Leu)	0.68	0.014
Histidine (His)	0.108	0.002
Arginine (Arg)	0.301	0.043
Lysine (Lys)	0.19	0.006
Aspartic acid (Asp)	1.511	0.052
Glutamic acid (Glu)	1.317	0.034
β-amino isobutyric acid	1.049	0.234
γ-amino-n-butyric acid	0.216	ND*
Serine (Ser)	0.591	0.020
Threonine (Thr)	0.326	0.008
Proline (Pro)	1.621	0.030
Tyrosine (Tyr)	0.503	ND*
Valine (Val)	ND*	0.039
β-alanine	0.618	0.0847
Ethanolamine	ND*	0.0356
Carnosine	0.193	0.1446
Hydroxyproline	0.685	0.0165
Total free amino acid	21.662	1.040

*ND:Not Detected

### The antibacterial effect of *Colla corii asini* toward *S. typhimurium*

**Fig. 1:**
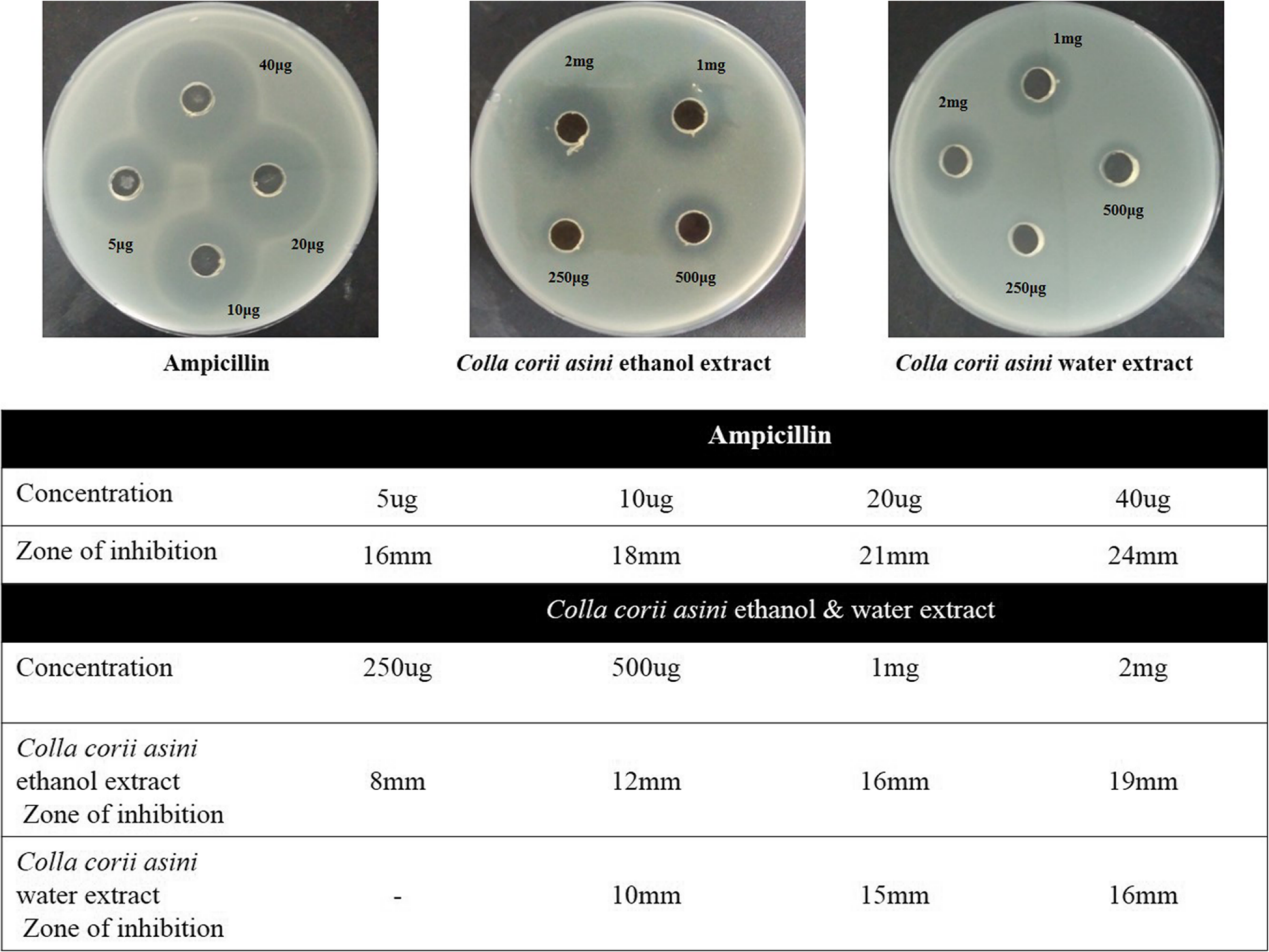
The agar well diffusion assay of *Colla corii asini* extract. We determined the zone of inhibition for *Colla corii asini* extract and the positive control (ampicillin) after 24 h. The zones of inhibition with the various concentrations of *Colla corii asini* ethanol and water extract were greater for *S. typhimurium*. The *Colla corii asini* ethanol extract had more of a significant effect than the *Colla corii asini* water extract

To investigate the antibacterial effect of *Colla corii asini*, we performed an agar well diffusion assay. The negative control (vehicle) did not show bacterial growth around the well (data not shown). The antibacterial effect was determined on the basis of the zone of inhibition around *Colla corii asini* extract and the positive control (ampicillin) after 24 h (Fig. 1). The zone of inhibition with the various concentrations of CEE and CWE increased in a dose-dependent manner for *S.* typhimurium. The CEE (19 mm) had more of a significant effect than the CWE (16 mm).

**Fig. 2:**
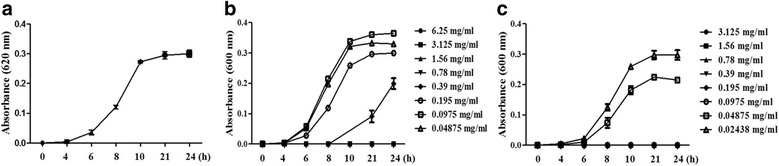
The growth curves of *S. typhimurium* treated with *Colla corii asini* extract. We quantitatively evaluated the potency of the Colla corii asini ethanol extract by determining the MIC. **a** The growth curve of *S. typhimurium* with vechicle. **b** The growth curve of *S. typhimurium* with *Colla corii asini* ethanol extracts. **c** The growth curve of *S. typhimurium* with ampicillin. We calculated an MIC value of 0.78 mg/ml by using the tube dilution method. The growth of *S. typhimurium* was reduced by treatment with the *Colla corii asini* ethanol extracts. We calculated an MIC value of 0.78 mg/ml by using the tube dilution method

The antibacterial effect of the CEE and its potency were quantitatively evaluated by determining the MIC and MBC. In this study, the growth of *S.* typhimurium was significantly inhibited by the ethanol extract, thus resulting in an MIC of 0.78 mg/ml, as determined by the tube dilution method (Fig. 2b), and the growth of *S.* typhimurium did not affect by vehicle (Fig. 2a). However, the growth of *S.* typhimurium with ampicillin inhibited at 0.0975 mg/ml (Fig. 2c). The MBC and MBC 50 values of the *Colla corii asini* ethanol extract was determined to be 1.56 mg/ml and 0.78 mg/ml**,** respectively (Table 2). The results suggested that a high dose *Colla corii asini* can have an antibacterial effect.

**Table 2:** MBC of *Colla corii asini* ethanol extract

*Colla corii asini* ethanol extract	Dilution rate	Colony number				Average	Mean
Control	10^7^	300	285	121	143	212.2	4.5 × 10^8^
	10^8^	58	45	98	73	68.5	
0.78 mg/ml	10^3^	55	42	30	70	49.2	4.9 × 10^4^
1.56 mg/ml	10^1^	20	40	50	60	42	39
3.12 mg/ml	10^1^	20	50	20	20	28	
6.24 mg/ml	10^1^	50	50	60	30	48	

### CEE inhibits salmonella invasion proteins and mRNAs in *S. typhimurium* strains

**Fig. 3:**
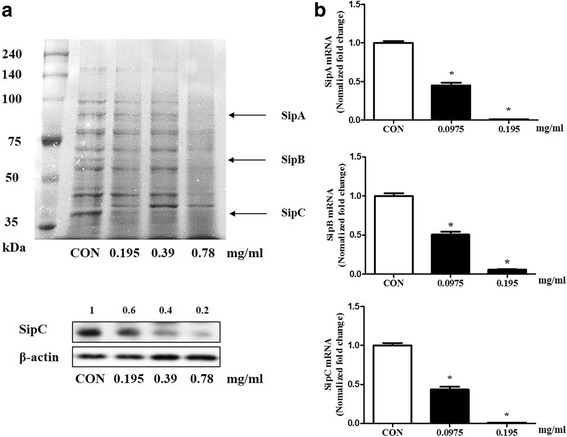
*Colla corii asini* ethanol extract inhibited the proteins and mRNAs of T3SS-1 effectors in *S. typhimurium.* We detected the secretion of T3SS-1 effectors in the bacterial supernatant by western blotting. **a** The *Colla corii asini* ethanol extract (0.195 mg/ml) significantly reduced the salmonella invasion proteins such as SipA, SipB and SipC. **b** We treated *S. typhimurium* with various concentrations of *Colla corii asini* ethanol extract. After 24 h, we extracted mRNA from *S. typhimurium* and conducted real-time PCR. *Colla corii asini* ethanol extract (0.195 mg/ml) significantly decreased the mRNA levels of salmonella invasion proteins

*S.* typhimurium triggers intestinal diseases via the invasion of the small intestines. During this process, salmonella invasion proteins including SipA, SipB and SipC play a critical role. To study the secretion of the effectors, we applied various concentrations of CEE to *S.* typhimurium 1 h before analysis. The MIC value was 0.78 mg/ml; however, a value of 0.39 mg/ml slightly reduced bacterial growth. We determined that the maximum concentration was 0.195 mg/ml (no effect on bacterial growth) (Table 3). Proteins were obtained from culture supernatants and bacteria, and then analyzed by SDS-PAGE and Western blot to evaluate the secretion of T3SS-1 effectors in the bacterial supernatant (Fig. 3a). The expression of bands with sizes 80 (SipA), 68 (SipB) and 40 (SipC) kDa was evaluated for the inhibition of invasion proteins of *S.* typhimurium. The concentration of 0.195 mg/ml (lane 2) reduced the levels of salmonella invasion proteins such as SipA, SipB and SipC. Especially, SipC protein were significantly reduced by CEE on Western blot analysis. Additionally, we investigated the effects of *Colla corii asini* ethanol extract on mRNA levels of *S.* typhimurium invasion proteins by using real-time PCR analysis. *S.* typhimurium was treated with various concentrations of CEE for 1 h and mRNAs of invasion proteins, including SipA, SipB and SipC, were analyzed. The mRNA levels were significantly decreased at a 0.195 mg/ml concentration (Fig. 3b). The data showed that low-dose CEE had an antibacterial effect via the inhibition of the expression of invasion proteins.

**Table 3:** Determined the maximum concentration of *Colla corii asini* ethanol extract (no effect on bacterial growth)

	Dilution rate	Colony number			Average	Total	Mean
Control	10^7^	19	85	50	52	5 × 10^8^	4.7 × 10^8^
	10^7^	41	43	46	42	4.2 × 10^8^	
	10^6^	354	497	614	488.3	4.9 × 10^8^	
*Colla corii asini* ethanol extract (0.195 mg/ml)	10^7^	47	56	60	54.3	5.4 × 10^8^	4.3 × 10^8^
	10^6^	325	323	320	323.0	3.2 × 10^8^

### CEE inhibits the invasion of *S.* Typhimurium in Caco-2 cells

**Fig. 4:**
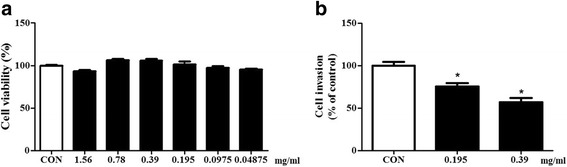
The effect of *Colla corii asini* ethanol extract on cell invasion of *S. typhimurium*. ***a*** *Colla corii asini* ethanol extract did not have an effect on Caco-2 cell viability. **b** We treated *S. typhimurium* with various concentrations of *Colla corii asini* ethanol extract. After 1 h, Caco-2 cells were washed three times with PBS, and gentamicin (100 μg/ml) was added for 1 h to kill any remaining bacteria. Caco-2 cell lysates were spread on solid agar. After 24 h, we counted the colony numbers. *Colla corii asini* ethanol extract (0.195 mg/ml) significantly inhibited *S. typhimurium* invasion in Caco-2 cells

We investigated the effects of CEE on cell cytotoxicity in Caco-2 cells. The Caco-2 cell viability was measured after treatments with various concentrations of CEE (1.56–0.04875 mg/ml) and incubation for 1 h. The CEE did not affect cell cytotoxicity (Fig. 4a). Also, we determined the LC_50_ value of Vero cells (derived from the kidney of an African green monkey) and calculated and presented in Table 4. Additionally, CEE significantly reduced the invasiveness of *S.* typhimurium in Caco-2 cells (Fig. 4b). The results suggested that low-dose *Colla corii asini* inhibited the invasion of the bacteria in vitro.

**Table 4:** Cytotoxicity (IC_50_ in mg/mL) of *Colla corii asini* ethanol extract and selectivity index (SI) values against Vero cells

	Antibacterial activity (MIC, mg/ml)	Cytotoxicity IC_50_ (mg/mL) Vero Cells	Selectivity Index IC_50_/MIC
	S. typhimurium		
*Colla corii asini* ethanol extract	0.78	4.75	6.09

### CEE attenuated the invasion of *S.* Typhimurium in mice intestines

**Fig. 5:**
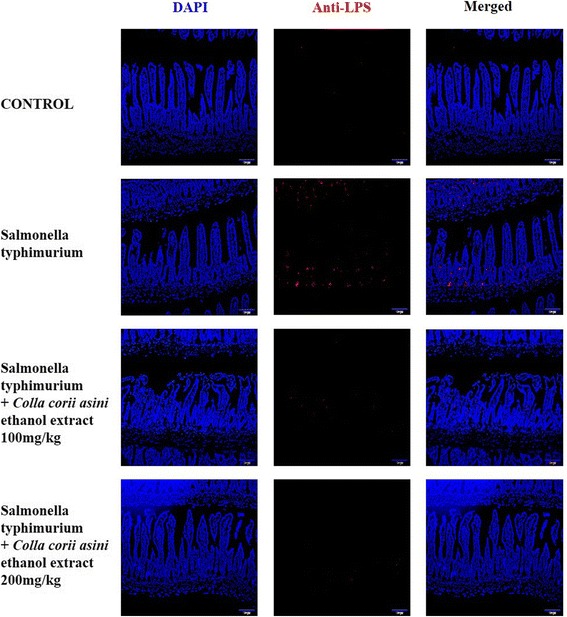
*Colla corii asini* ethanol extract inhibited the invasion of *S. typhimurium* in mice intestines. ICR mice were orally infected with *S. typhimurium* and the small intestines were obtained. Serial sections of intestines were analyzed via IHC. *Colla corii asini* ethanol extract (200 mg/kg) suppressed the invasion of bacteria compared with that in the group of mice treated with *S. typhimurium* only

To evaluate the function of CEE during a host infection by *S.* typhimurium, ICR mice were orally infected with *S.* typhimurium. The “Swiss rolled” tissues were divided into 5 μm sections, and IHC was performed. The CEE attenuated the invasion of bacteria in a dose-dependent manner. Specifically, mice receiving the CEE treatment (200 mg/kg) had suppressed bacterial invasion, as compared with the group treated with *S.* typhimurium only (Fig. 5). The results suggested that low-dose *Colla corii asini* inhibited the invasion of bacteria in mice.

## Discussion

*S.* typhimurium is the main cause of gastroenteritis, and it occurs via bacterial gastrointestinal infections from contaminated food, thus causing various diseases and enormous economic loss. Normally, *S.* typhimurium-related diseases occur when bacteria penetrate the gastro-intestines [24]. Despite the growing recognition of hygiene and health, salmonella infections have increased worldwide, and the underlying mechanisms remain unclear. Additionally, mutant bacteria and antibiotic resistance has increased, and new therapeutic agents without side effects are needed [25].

*Colla corii asini* (E‘jiao) is prepared from the skin of *Equus asinus* and is used in traditional Chinese medicine and nutritional supplements in China and Korea [2]. We conducted experiments to determine the antibacterial effects of approximately four hundred kinds of Korean medicine on *S.* typhimurium, and *Colla corii asini* was the most effective. We hypothesized that *Colla corii asini* had an antibacterial effect through killing *S.* typhimurium as well as inhibiting the invasion of the host.

In the present work, the extracts obtained from *Colla corii asini* showed strong activity against *S.* typhimurium. Our data showed that CEE at a high dose (the inhibition of bacterial growth) possessed an antibacterial effect. An agar well diffusion assay and MIC/MBC values indicated that *Colla corii asini* might be the new antibacterial agent (Figs. 1 and 2, Table 2).

In previous studies, human and animal models of salmonella infection have investigated many TTSS effector proteins such as SipA, SipB and SipC and have shown that they are involved in inflammatory responses and diarrhea [26]. Salmonella invasion proteins play a pivotal role in the infection process and facilitate bacterial invasion in non-phagocytic cells in the host [27, 28]. SipA is an important protein promoting salmonella invasion [29]. SipB is a substrate of the SPI1 type III export system [30] and deficient bacterial SipB do not enter eukaryotic cells [27]. SipC is a protein with diverse functions, and its actin nucleation and bundling activities play a critical role in salmonella-induced actin cytoskeleton reorganization and bacterial invasion [31]. Our data showed that CEE at low doses (no effect on bacterial growth) significantly decreased the expression of salmonella invasion proteins including SipA, SipB and SipC in *S.* typhimurium. Additionally, the mRNA expressions of salmonella invasion proteins were suppressed in a dose-dependent manner (Fig. 3a and b). To confirm the effect of CEE on salmonella invasion, we conducted gentamicin assays. CEE did not affect Caco-2 cell viability and attenuated the bacterial invasion in the Caco-2 cell monolayer (Fig. 4a and b). Furthermore, CEE inhibited the invasion of *S.* typhimurium in the small intestines (Fig. 5).

## Conclusions

Our findings indicated that *Colla corii asini* contains potential antibacterial effects that may be a new pharmaceutical drug for treatment of *S.* typhimurium-related diseases. The ethanol extracts of *Colla corii asini* exert significant inhibitory effects against *S.* typhimurium via the inhibition of bacterial growth and cell invasion. The results of the study provide clues to understanding the antibacterial action of *Colla corii asini*.

## Abbreviations

CEE: *Colla corii asini* ethanol extract
MBC: Minimum Bactericidal Concentration
MIC: Minimum Inhibitory Concentration
*S.* typhimurium: *Salmonella enterica serovar* Typhimurium
